# Exploring the social context of smoking behaviours: insights from stop-smoking advisors in deprived communities in Northwest of England UK

**DOI:** 10.1186/s12889-025-23110-7

**Published:** 2025-05-23

**Authors:** Mzwandile Mabhala, Winifred Adaobi Esealuka, Asmait Yohannes, Amanda Nkolika Nwufo, Lahja Paulus, Meron Tefera, June Keeling

**Affiliations:** 1 University of Derby, School of School of Allied Health and Social Care, Derby, DE22 1GB UK; 2 St James Hospital, Beckett St, Harehills, Leeds, LS9 7 TF UK; 3 Mount Sinai Health System, 150 East 42 Nd Street, Second Floor, New York, NY 10017 USA; 4 Bulwell Riverside, Main St, Bulwell, Nottingham, NG6 8QJ UK; 5 The Walton Centre NHS Foundation Trust, Lower Lane, Fazakerley, Liverpool, L9 7LJ UK; 6 Stoke Mandeville Hospital, Mandeville Road, Aylesbury, HP21 8 AL UK; 7 School of Nursing and Midwifery, David Weatherall Building, Keele University, Stoke-On-Trent, ST5 5BG UK

**Keywords:** Stop-smoking, Disadvantaged communities, Social context, Community engagement

## Abstract

**Background:**

Successive UK governments have introduced measures to enhance access to stop-smoking services. However, these efforts have primarily focused on restricting access to tobacco products and promoting individual behaviour changes, overlooking the social conditions that contribute to smoking behaviours. While promoting individual behaviour changes can be beneficial, lasting change requires collective action and structural reforms. This research examines the limitations of individual-focused stop-smoking interventions in deprived communities. It emphasises the significance of adopting a comprehensive smoking cessation strategy that take into account the broader socioeconomic determinants. These findings are crucial for understanding the complexities of smoking cessation in deprived communities.

**Methods:**

This study uses interpretive phenomenology and socioeconomic determinants theories to analyse the experiences of stop-smoking advisors in promoting smoking cessation initiatives within a disadvantaged neighbourhood in northwest England. In this study, stop-smoking advisors are practitioners trained to provide support and guidance through various methods, such as one-on-one counselling, group meetings and workshops. The research took place between March and July 2019 at a local authority-owned lifestyle centre in the most deprived community in northwest England.

**Results:**

The analysis identified four overarching themes: 1) Developing a skilled, confident, and culturally competent stop-smoking advice team, 2) Understanding other complex social, mental, and physical health issues, 3) Bringing the stop-smoking programme to those who need it the most, 4) Adapting the service to meet the user’s needs.

**Conclusions:**

The behaviour-oriented interventions have resulted in a disproportionate decrease in smoking rates, with a more rapid decline in the least deprived areas compared to the deprived ones. The inverse care law theory provided a compelling framework for understanding these differences. It emphasised equal consideration of behavioural and structural interventions in addressing smoking habits in deprived neighbourhoods, highlighting the impact of socioeconomic factors and the limitations of individual behaviour-focused stop-smoking interventions.

**Supplementary Information:**

The online version contains supplementary material available at 10.1186/s12889-025-23110-7.

## Introduction

In the United Kingdom (UK), the net smoking prevalence is continuing to decline yearly [1–9], with 11.9% people who smoke in 2023 [9] compared to 16.9% in 2015 [1]. However, the decline in smoking prevalence is not equally distributed across all population groups [10–13]. It remains significantly higher among socially disadvantaged groups [1–10, 14, 15].

Table 1 indicates that although all groups have seen a decline in smoking prevalence, the decrease was more significant among those in managerial and professional positions compared to individuals in manual and routine jobs.

**Table 1:** Percentages of smokers by socioeconomic indicators: employment status of people aged 18 years and over in the UK between 2015 and 2023

		**Smokers (%)**	**Smokers (%)**
Year	Overall % smokers	Routine workers	Managerial and professional
2015	16.9	30.0	9.0
2016	15.8	29.1	7.6
2017	15.1	25.9	10.2
2018	14.7	25.5	10.2
2019	14.1	23.4	9.3
2020	13.8	Not reported	Not reported
2021	13.3	28.2	6.6
2022	12.9	22.8	8.3
2023	11.9	20.2	7.9

Source: The data in Table 1 is extracted from reports by the UK Office of National Statistics between 2015 and 2023

The UK governments, particularly from 2002 to 2023, have implemented policy initiatives aimed at making tobacco and vaping products less attractive and limiting the availability of tobacco products [16–20]. Some of the policy initiatives they implemented include legislation restricting tobacco advertising [18], introducing standardised packaging [19], and banning menthol-flavoured tobacco products [20]. Furthermore, they launched the “Swap to Stop” campaign, which aims to encourage people who smoke to transition to vaping [12], invested over 70 million pounds annually to support local authority-led stop-smoking services (SSS) and introduced the Tobacco and Vapes Bill 2024 to create the first-ever smoke-free generation [17].

However, the government’s tobacco control policies appear to have primarily focused on limiting access to tobacco products and encouraging individual behaviour change, with little consideration given to the social conditions that contribute to smoking behaviours. These measures resulted in greater benefits for the least deprived compared to the most deprived, reflecting an average decline in smoking of 26.4% among the least deprived, while the most deprived experienced an average decline of only 8.7% [1–9]. Numerous studies have shown that behaviour-oriented interventions may be less effective in disadvantaged populations [14, 21–27]. This observation aligns with Tudor-Hart’s inverse care law, which states that the availability of high-quality health and social care services often decreases as the need for those services increases in the population being served [21]. Therefore, it is crucial to acknowledge the social context of smoking to develop effective stop-smoking interventions and reduce the prevalence of smoking in all segments of society [14, 22–26, 28, 29].

While promoting individual behaviour changes can be helpful, it is important to recognise that lasting change requires collective action and structural reforms. In the face of growing health inequalities [30–32], solely focusing on individual behaviour changes tends to overlook the significance of structural factors, social processes, and local settings affecting people’s health and ability to adopt healthy lifestyles.

The social determinants of health (SDH), the fundamental cause theory (FCT), the political economy approach, and the eco-social theories provide strategic entry points for policy action to address the structural determinants of smoking behaviour [33]. They offer some insight into the effect of local community-level deprivation on smoking behaviour [33]. They indicate that many individual lifestyle behaviours reflect adverse social conditions in which people are born and grow up [34, 35]. Considering these insights, incorporating wider socioeconomic determinants of health into smoking cessation—such as education, employment, income, housing, environment, crime prevention and health, would effectively address not only smoking behaviours but also other health-harming behaviours [34, 36].

Many studies have built upon these theories to illustrate the impact of a neighbourhood’s level of deprivation on smoking behaviour [14, 22, 23, 25, 26]. They argue that the neighbourhood plays a significant role in determining the likelihood of being a person who smokes and the challenges one may face in quitting, notwithstanding their socioeconomic status [22, 24, 37–40]. For example, an Australian study by Turrell et al., in line with research from the UK [14] and the US [38, 39], reported that after adjusting for individual differences in occupation, education, income, gender, and age, people living in the most deprived communities were less likely to quit smoking (9.3–12.5%) than more affluent individuals (23.1–25%).

Galster’s [41] study explains the theoretical connection between communities and individual outcomes, proposing theoretical mechanisms to aid in smoking cessation at the neighbourhood level. According to Galster, the mechanisms of neighbourhood effect theory, including social-interactive, environmental, geographical, and institutional factors, play a role in initiating and sustaining smoking in deprived communities [41]. Social-interactive mechanisms are based on the idea that socially disadvantaged communities create conditions that increase the likelihood of initiating smoking and reduce the likelihood of quitting [24, 26, 41, 42]. These conditions include poor job prospects, as some employers are reluctant to consider people from ‘bad areas’ [26, 41]. In addition, disadvantaged communities provide fewer opportunities for social capital, participation, and interaction with employed individuals [26, 41, 43]. Furthermore, the environment where disadvantaged people live tends to increase exposure to stresses produced by unemployment, lack of basic amenities, higher crime, violence, and incivilities such as littering and vandalism [41]. The evidence indicates that communities experiencing these adverse social conditions often turn to maladaptive coping mechanisms, such as smoking [43, 44]. Several qualitative studies have reported that people who smoke from the deprived neighbourhood face unique barriers to quitting [45–48]. These included low motivation to quit, high anxiety/boredom, normalisation of smoking and widespread illicit tobacco use [45–47].

Galster proposes that disadvantaged communities’ geographical location and poor infrastructure create physical and social disconnection from neighbouring more affluent suburbs and larger urban areas [24, 26, 49–51]. The poor infrastructure means that if they find employment, they will likely face greater transport and childcare difficulties [26]. Considering the impact of neighbourhood deprivation on smoking habits, it would be logical for the government to give equal consideration to individual behaviour initiatives and the structural factors contributing to neighbourhood deprivation.

Having examined the evidence that demonstrates the unequal distribution of smoking among various population groups, this article argues that socioeconomic deprivation is a fundamental determinant of smoking behaviour. We have also examined the government’s efforts to reduce smoking rates and assert that the policies primarily focus more on promoting individual behaviour change than wider socioeconomic determinants of smoking behaviour. We outlined the limitations of individual behaviour-focused stop-smoking interventions in deprived communities. This study aims to use interpretive phenomenology and socioeconomic determinants theories to analyse the experiences of stop-smoking advisors in promoting smoking cessation initiatives within a disadvantaged neighbourhood in northwest England. This approach will provide a unique theoretical understanding of how the neighbourhood affects smoking behaviour. In this study, stop-smoking advisors are practitioners trained to provide support and guidance through various methods, such as one-on-one counselling sessions, group meetings, and educational workshops.

## Method

The interpretive phenomenology [52] and socioeconomic determinants theories [34, 53–55] were used to analyse the data collected by MM and EWA regarding the experiences of stop-smoking advisors in promoting smoking cessation initiatives within a disadvantaged neighbourhood in northwest England. This study incorporated a reflexive component, in which the researchers articulated how their specialised knowledge of the socioeconomic determinants of health inequalities and their philosophical beliefs in social justice principles influenced all facets of the research project.

The research took place between March and July 2019 at a local authority-owned lifestyle centre in the most deprived community in northwest England.

The university and local authorities collaborated, allowing a second author (postgraduate research student) to spend one day a week (between March and July 2019) with the local authorities to observe the stop-smoking advisors and learn from their experiences.

### Setting and recruitments

The stop-smoking service is set in one of the most disadvantaged lower super output areas in Cheshire, with an unemployment rate of 4.6%, almost double the borough’s 2.2% [56–59]. Additionally, 34.3% of the population is economically inactive due to retirement, disability, or caregiving responsibilities, and 38.5% of residents do not have any qualifications [56–59]. Super Output Areas (SOAs) are a geographic framework designed to improve the reporting of small area statistics in England and Wales [60]. There are three layers of SOAs: Lower, Middle, and Upper, each with consistent sizes and stable boundaries [60]. Lower Layer Super Output Areas (LSOAs) are small areas that typically contain an average of 400 to 1,200 households, with a resident population ranging from 1,000 to 3,000 individuals [60].

The recruitment strategy was based on ethical principles of voluntary participation and equal opportunity to participate. To achieve this, we emailed each stop-smoking advisor from the participating services and invited them to take part. The participating service consists of six stop-smoking advisors, all of whom were invited, agreed to, and available to participate at the requested times.

### Data collection

Data collection methods consisted of individual face-to-face interviews, observations and reflective diaries. The researchers maintained a reflective fieldwork diary to record their observations and reflections. After three months, in-depth one-to-one semi-structured interviews were conducted with the stop-smoking advisors (*n* = 6). Aligned with phenomenology, the analysis and findings focused on participants’ descriptions of their experiences rather than on observations.

The senior researcher and researcher conducted the one to one in person interviews, each lasting between 45 and 60 min. Interview guide was used to ensure that same questions were asked in all participants (Interview guide in appendix 1). This was specifically developed for this study, and it has not been used in any previous studies. The interviews were audiotaped. The audio tapes were transcribed verbatim by the researchers.

The interviews focused on understanding stop-smoking advisors’ experiences delivering stop-smoking programs in deprived communities. The data presented in this study comes from the analysis of the reflective diary and transcripts from 1:1 interview with the stop-smoking advisors.

### Ethics approval

The study received ethical approval from the University of Chester’s Research Ethics Committee. The committee carefully reviewed the study design, research materials, and participant information sheet. The participant information sheet included a letter of invitation highlighting the voluntary nature of participation. All potential participants were given a detailed information sheet containing information about the study, interview questions, and data usage. Participants had seven days to decide whether they wanted to take part. Those who agreed to participate signed a consent form. All participants consented to publish their combined data without including any identifiable information.

The centre managers granted access to the participants and premises, respecting participant privacy. Pseudonyms were used to maintain participant anonymity. The name of the local authority is not disclosed, demonstrating the commitment to complying with the anonymity requirement of the ethics committee.

### Data analysis

The analysis drew on Benner’s [52] principles of interpretive analysis. Consistent with interpretive phenomenology, we aimed to examine the meanings stop-smoking advisors ascribe to experiences of being in their world of smoking cessation services [52]. Therefore, we considered interpretative phenomenology philosophy the most appropriate for our research aim. Two aspects of interpretative phenomenology influenced the design of this study [52]. The first is the concept of ‘being in the world’ [52]. By this concept, Heidegger postulated that the best way to understand people is to understand their world [52]. Through the notion of being in the world, we sought to understand the practices that stop-smoking advisors have by virtue of being in context. The second aspect of that was consistent with the aim of this study is the epistemological assumption that people talk about the essential experiences that are meaningful to them in their contexts. Therefore, our task as researchers is to discover the meanings that participants ascribe to their being in the context.

**Fig. 1:**
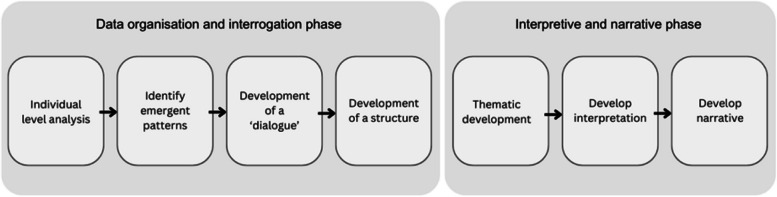
illustrates the two phases of interpretive qualitative data analytic process used to analyse the data collected from March to July 2019 in Northwest England

The collected data was organised according to the thematic analytic process outlined by Benner [52]. The data analysis was conducted using a Microsoft Word document, without the use of any computerised analytical software. The transcribed data was reviewed on-screen, and the Track Changes feature was utilised to make margin notes that highlighted significant theoretical incidents in the data. The data analysis was broadly organised according to the two phases of interpretive phenomenological analysis—data organization and interrogation and interpretive and narrative phase -a process summarised in Fig. 1.

### The data used to populate this figure one is extracted from Larkin and Thompson [61]

As demonstrated in Fig. 1, the interpretive and interrogation phase began with a line-by-line analysis of individual participants’ experiences within a specific context. We identified the emerging patterns of meaning that participants ascribed to their experiences. These experiences were then coded using concepts that captured the patterns observed. Following this, we explored the relationships among the different themes, posing questions about the significance of participants’ concerns within this context. Next, we developed a structural fra*mework* to illustrate the relationships between the themes. The second phase involved clustering themes with similar meanings into overarching themes and subthemes, along with providing theoretical explanations for these groupings [61].

## Results

Table 2: below shows the characteristics of stop-smoking advisors who participated in this study.

**Table 2:** Stop-smoking advisors involved in a study exploring the social context of smoking behaviours in deprived communities in the Northwest of England, UK, between March and July 2019

Advisors	Sex	Stop-smoking-specific experiences	Overall working experience
Alice	Female	7 years	30
Beth	Female	17 years	17
Carl	Male	10 years	15
Danielle	Female	19 years	19
Ellen	Female	6 years	10
Fran	Female	17 years	20

### Pseudonyms have been used for anonymity

The analysis revealed four themes and corresponding subthemes (see Table 3). These explain how this service is organised and delivered.

1. Developing a skilled, confident, and culturally competent stop-smoking advice team.
2. Understanding other complex social, mental, and physical health issues.
3. Bringing stop-smoking programmes to those who need it the most.
4. Adapting the service to meet the user’s needs.

**Table 3:** The overarching themes and corresponding subthemes illustrate how the Lifestyle Centre stop-smoking programme is organised and delivered

Overarching theme	Subthemes
Developing a skilled, confident, and culturally competent stop-smoking advice team	Having accreditation as a stop-smoking advisor
	Draw from a wide range of backgrounds
	Having extensive caring experience
	Understanding different cultures
Understanding other complex social, mental and physical health issues	Working with them on overall lifestyle
	Understand their social circumstances
	Knowing a person as a whole People not prioritising smoking cessation
Bringing stop-smoking programmes to those who need them the most	Stop smoking service is integral to the NHS patient’s healthcare package
	Rethinking the indicators of successful stop-smoking services among young people
	Schools: Shifted from intense programmes for children and young people to minimum harm-reduction measures
	Prisons: delivering nationally recognized and award-winning work
Adapting the service to meet the user’s needs	Offering flexible access
	Recognising that people progress differently
	Designing service around users’ circumstances
	Matching the intervention with the right user
	Establish the motive to stop smoking

Stop-smoking advisors in this study highlighted the challenges of delivering smoking cessation services within deprived communities and the limitations of generic individual behaviour-oriented approaches.

### Developing a skilled, confident, and culturally competent stop-smoking advice team

The study’s advisors recognise that addressing the unique and complex needs of people who smoke from deprived communities requires more than just generic stop-smoking skills. They identified several elements that they believe are unique and useful to their role as smoking cessation advisors, both individually and collectively as a team:

1. Having accredited training as stop-smoking advisors.
2. Being drawn from a wide range of backgrounds.
3. Having extensive caring experience.
4. Understanding different cultures.

The advisors had to undergo extensive training to develop competencies and accreditation as a stop-smoking advisor. Beth described how this training was crucial.

*Beth: I did all the in-house training, some motivational, CBT, motivational interview and that kind of stuff. That was how I got to the role and applied for the job as the stop-smoking assistant and then the stop-smoking advisor. Then I went to London and did the Maudsley Smoking Cessation Training. I have built up my training like that. Recently, I got the National Centre for Smoking Cessation Training (NCSCT) online training, which makes me officially a smoking cessation practitioner.*

The advisors drew on their extensive experience in working with people. For instance, Alice explained that she had thirty years of experience in caregiving roles within the NHS, including dealing with childhood physical and mental illnesses. Her work and academic experiences led to her current position.

*Alice: my NHS career started over 30 years ago; originally, I was in child health, so working within the medical facility for child health as it was then in [the Northwest of England]. From there, I went into child health and child psychiatry, all in admin support roles, and then I went into continuing care. So, from the continuing care role, I then progressed to GP practice and from that, I combined health and social care qualifications with the Open University and then saw an opening; it wasn’t lifestyles; it was still under the NHS umbrella, for a secondment and training to become a stop-smoking advisor.*

Similarly, Beth suggests that having experiences in a wide range of settings adds to her approach to stop smoking, implying that she draws upon a wide range of experiences.

*Beth: I’ve probably been doing this job for about seventeen years now, so I’m working across the community, doing a lot of work with young people in schools. When I started my job, we were with health promotion, so I’ve seen all the changes from health promotion to primary care trusts (PCT) and local authorities.*

The neighbourhood has a large population of migrant people who smoke. Advisors believe that understanding the culture of the people they work with is essential for the success of their program. Two of them have experience working abroad, which is advantageous in engaging with some of the seldom heard groups in migrant communities. They bring knowledge of working with non-English-speaking people.

*Carl: I think it’s important to engage with different cultures or even people who don’t speak English as a first language; that’s never a barrier to me because I worked in …, so I had to work with people who couldn’t even speak English, so it was good for me, particularly around the culture…*

The advisors’ adaptability and experiences working with diverse populations, including migrants, contribute to the success of the stop-smoking programme. Understanding and transcending cultural barriers are seen as essential for effectively engaging with seldom heard groups, emphasising the significance of cultural competence in smoking cessation efforts within deprived communities.

### Understanding other complex social, mental and physical health issues

All advisors observe that people who smoke have wider issues, such as complex mental health issues, social isolation, poor housing, low literacy, and poverty, rather than focusing on smoking behaviours. Carl describes a typical people who smoke they deal with regularly.

*Carl: So, we’re dealing with people who relapse, constantly relapse, so and because the smoking prevalence has been reducing, and we’re beginning to see more complex clients. So, we’ve also had to change how we deal with these people.*

The advisors criticise the idea that merely providing information will motivate individuals to change their smoking habits. They believe that without addressing social conditions, a smoker will struggle to quit.

*Danielle: I mean, we can keep trying to encourage them to see. They know they would be better off, say, in finance. People who have medical conditions know that they would be better off if they weren’t smoking.*

Confirming the assertion that the lack of engagement with stop-smoking services among people from deprived communities is not due to a lack of knowledge about the harmful effects of smoking. Advisors interacting with people from these communities concluded that individuals who smoke are aware of the harmful effects of smoking but continue to smoke to cope with their adverse health and social conditions. Therefore, advisors proposed integrating stop-smoking services into other population-based interventions.

*Ellen: They know smoking is bad for them but do not understand why it’s so bad. Some of our roles here as a team are to do less of the traditional smoking offer and more of the education around helping people understand why. We’ve started working from the individuals’ circumstances… their mental health, home life, housing situation and how they spend their days.*

The advisor elucidates that certain individuals present with a range of health-compromising behaviours, some of which necessitate more immediate intervention than smoking cessation. The advisor provides examples in which, while intending to offer smoking cessation guidance, they recognised the presence of urgent mental health conditions and issues related to excess alcohol use that required prioritised attention.

*Danielle: I had a lady last week. She has mental health problems, and she had clearly been drinking as well and was constantly talking about drinking vodka when she went back, so it’s like, so in the grand scheme of things, quitting Smoking, you know, I think well, shouldn’t we be tackling the vodka issue first? And then she was talking about all other mental health problems. She also talked about the range of problems she has with her daughter’s mental health. As smoking cessation advisors, we had to abandon all that to refocus the conversation to stop smoking.*

These incidents made it necessary for advisors to receive training for the extended roles, including mental health first aid, suicide awareness, safeguarding, and referral skills. This training allows them to deal with mental health issues within their scope of practice and refer complex cases to specialist services.

*Ellen: So, we do extended training like mental health first aid, suicide awareness, signpost to the health and wellbeing team at lifestyle centre, so we feel like we know where to access information, and if the team don’t, they tend to come to me for that information. So, we feel equipped, but we could do more. So, we have the safeguarding side covered because … we have policies and procedures in place.*

*One example of safeguarding: a couple of weeks ago, we had a young person who was eight years old, his brother was eleven, and his eldest was fourteen and the mum had brought them in cos they needed to stop Smoking; she didn’t identify that they were getting the cigarette from their peers. We brought the case to the team meeting, discussed it and created a plan.*

Advisors suggest that smoking cessation interventions should focus on overall lifestyle changes, not just quitting smoking.

*Alice: In an ideal world, you would like to pull it right back before somebody starts on a lifestyle change and start with their self-esteem and confidence. Isolation is such a massive, massive thing.*

Advisor claimed that the combination of low esteem, social, physical and mental health problems and lack of work limits their capacity to engage in healthy behaviour. Advisors believe that tackling the fundamental cause would be more beneficial than traditional stop-smoking approaches for such people.

*Alice: Yeah, and I think the people we see now are hardened smokers with many other issues. For any lifestyle change, not only Smoking, but I also feel it’s so important to get the foundations right first because many people (and I’m not making judgements here), their esteem is on the floor, they’re not always working, they’ve got lots of other problems either themselves, extended family and not always the energy. It is hard enough to manage with one condition if you’ve got several comorbidities.*

The model for behavioural change in smoking cessation emphasises that providing knowledge about the health risks of smoking can lead to significant behavioural change. This study confirmed that people who smoke in disadvantaged communities face significant challenges that may prevent them from making healthy choices. These challenges include issues such as housing instability, social isolation, mental health struggles, and financial difficulties.

Stop-smoking advisors observed disparities between government priorities and the needs of the local population. For instance, it emerged in this study that when the public was consulted about local funding priorities, it became clear that there were differences in opinion. While the government prioritised improving access to smoking cessation programmes, the public preferred allocating funds to park development.

### Bringing stop-smoking programmes to those who need them the most

The advisors posit that smoking will not be eradicated as long as health inequalities exist.

*Ellen: These issues may not be completely eradicated regarding health inequalities, probably because people are forced by their circumstances to make lifestyle choices regarding smoking and relapsing.*

They explain that to overcome the neighbourhood effect on smoking prevalence, the local government is setting up smoking cessation services within the most deprived lower super output areas (SOA).

*Danielle: they try to put many of their [stop-smoking promotion] sessions in the SOA areas so they can access them easily because they’re the people we want to help the most. They’re the people who don’t access the service, so it’s trying to make it as accessible as possible. It’s trying to stay local to those deprive areas.*

To enhance community engagement with stop-smoking services in underserved areas, local authority stop-smoking commissioners allocated significant funding to one of the deprived communities to address health priorities and minimise health inequalities. They set aside some funding to promote community involvement in stop-smoking services. Importantly, the commissioners encouraged the communities to suggest their own interventions for reducing health inequalities rather than imposing a top-down approach. The feedback was clear: Community members indicated that smoking cessation was not a priority in their neighbourhoods.

*Ellen: So, we approached [Area A] because it is our area with the biggest pockets of deprivation and the highest smoking prevalence of around 38%. They had funding from the housing company in the area, and their council tax was also reduced to help reduce the health inequalities in the area. We asked the community what they wanted and what they prioritised as their biggest health need; smoking didn’t come up high. The community decided they’d rather spend the funding on things like swing parks and activities rather than the health and wellbeing of the population.*

*So, it’s that challenge where you try to promote smoking cessation when they don’t identify as having a problem until they are diagnosed with a smoking-related disease.*

Advisors deliver stop-smoking advice in traditional healthcare facilities such as GP practices, mental health, and NHS acute hospitals and non-traditional healthcare facilities, including prisons, schools, councils, and lifestyle centres. The goal is to bring stop-smoking services to the neighbourhoods that need it the most.

They found that when promoting stop-smoking services within the NHS facilities, service users see them as part of the healthcare service and readily engage with them. Furthermore, advisors found information sharing easier, as NHS teams proactively refer their patients to them.

*Danielle: If we are within an NHS facility, we have NHS staff identity; in the GP surgery, we have more access. Most advisors have, but generally, we would have access to the NHS computer systems in surgery. It is easier to share information that way because we are all NHS staff.*

Some GP surgeries have integrated referral systems, whereby stop smoking service is integral to their patient’s healthcare package.

*Alice: In some GP practices, when new patients register, the staff tell them, we will work with you to look after your healthcare and lifestyle. So, we would like you to engage with stop-smoking advisors.*

Patients readily engage with them if they believe it’s part of GP service.

*Alice: [Users] always pick up if I’m ringing on the GP practice landline.*

People who smoke are more likely to use the service if the clinical staff refers them.

*Alice: Many of my sessions are GP practice-based, which works well. They know and respect the opinion of the GP and clinical team members. So, being in practice is a striking iron while it is hot.*

Their efforts in schools and with young people align with government policy, which targets achieving a hundred quits per calendar year among young individuals. However, they face several challenges. Firstly, designated funding is essential to meet this target.

*Ellen: We have a target of 100 young people quitting, which we have yet to achieve and haven’t for the last decade because many of our services have had funding cuts.*

Secondly, advisors have found it more difficult to help young people quit compared to adults, often focusing on harm reduction due to the lack of funding, which limits the service to schools with a higher prevalence of people who smoke.

*Alice: We’re doing less than we used to. In the past, we would go into schools and do sessions with the pastoral care teams. Currently, one of our advisors is doing one of the high schools because it has high numbers of smokers. We did a while with a few smokers in one of the other schools, so we supported more harm reduction.*

Additionally, the guidelines suggest that individuals who do not quit within a certain period should be removed from the programmes. However, advisors note that it takes longer to convey the message to younger individuals who are still experimenting with tobacco.

*Ellen: I always feel real strongly about young people that if a young people come to us, we’re not going to get a four-week to quit from them because they’re still learning about quitting; this may be their first experience of stopping smoking; and it’s really hard for young people to stop smoking. At this age, they are still experimenting and learning what works and what doesn’t. So, if we’ve managed to get them to cut down, we can measure them for harm reduction rather than a complete quit.*

Therefore, they believe that measuring harm reduction is a more suitable indicator of success among young people than solely focusing on the number of quits.

*Danielle: It’s harm reduction, which should be measured. That way, there is a thing we can tick on the database because young people, generally, not all of them, but most don’t quit. They’re very difficult age groups, so it is harm reduction if you can get some to reduce the amount of smoking.*

The advisors have also successfully implemented a stop-smoking program within prison facilities. The primary objective of this initiative was to train prison staff to deliver the service to prisoners, ensuring its sustainability. This initiative’s success led it to become a national flagship, and one of the advisors was even nominated for a national award for their work in prisons, indicating success in this area.

*Alice: The idea is that we go in, speak to the staff, and try to get very brief advice on interventions so that we can do this in any setting. Then our plan is to do a pilot so that somebody can shadow and take over that role to keep continuity, which sounds great in reality.*

*Beth: [one of our advisors] was nominated for an award for the work he did in the prison... did some really good work with the prison*

### Adapting the service to meet the user’s needs

The stop-smoking programme offers a flexible access designed around users’ circumstances including face-to-face consultations, telephone consultations, and text messaging. Face-to-face consultations offer a distinct advantage, enabling comprehensive discussions and carbon monoxide testing to verify the quits. This personal interaction fosters a deeper understanding of the individual’s needs and enhances the programme’s effectiveness, ensuring that each individual’s unique circumstances are considered.

In their commitment to inclusivity, the advisors strive to provide the same quality of service regardless of the mode of delivery. They recognise the limitations of telephone consultations and text messaging, which are available for some potential service users who cannot attend face-to-face consultations due to various social, economic, and health reasons. To overcome these limitations, the advisors offer all new service users a half-hour initial consultation and subsequent weekly fifteen-minute face-to-face consultations for existing service users who can attend or a fifteen-minute telephone follow-up or text messages for those who cannot attend in person. This flexible approach ensures that all service users can access the programme and receive the support they need regardless of their circumstances.

*Danielle: if they’re a new person to the service, they have a longer appointment, just like the half-hour slot they’re booked into. If they’re a regular, it’s just a fifteen-minute- follow-up telephone appointment.*

Advisors aim to deliver consistent quality advice to all service users. Advisors explain that the quality of advice service users receive is the same regardless of the access mode.

*Danielle: … I am actually on telephone support, so that is obviously for the people who cannot attend for work or whatever commitments, so they are just booked into slots. We work our way through the list of people on telephone support. So, they still get the same kind of advice, and we still sort out prescriptions; it is just all done over the phone or by text while obviously in the one-to-one appointments is more in-depth, and also, we do a carbon monoxide testing so that is what we do when we see people face to face as well.*

The advisors closely monitor service users’ progress through the programme. They were cognisant that people progress at different paces. Some successfully complete the programme within twelve weeks, and others may take longer or even relapse. They encourage those who succeed to remain smoke-free and those who relapse to return to the service and start their journey all over again.

*Danielle: … people will come every 2 weeks, or they will ring every 2 weeks; in between that, we would send text messages to say did you get your prescription, any problems, let us know so that that goes on right through till they finish the course which for some people they’re on products for 12 weeks or some people midway through it will just disappear, and it is trying to get a hold of them to find out what happened to them so we have like a system where we will try and ring them a couple of times, we might even send them a letter.*

*If we get a hold of them and they say oh no, we are back to smoking, then they have relapsed and then invite them back. If they have successfully ended the program, they are usually followed up by phone call or text; it is 6 months and 12 months to see what happened to them once they have left.*

The advisor revealed that establishing the motive and readiness to stop smoking is essential for a successful quit. They learned from their experiences that smoking-related health crises could trigger people who smoke to seek help from stop-smoking services. The advisors understand that some may want to quit due to health crises but need conducive social and personal circumstances to sustain smoking abstinence. Under such circumstances, the advisors begin by building a productive relationship with the user, establishing the motives for wanting to quit, and making a full assessment of social and personal circumstances. The advisors endeavour to ascertain other habits around the users’ lives and gain insight into how they spend their days.

*Fran: So, if somebody rang in this morning and said the GP had referred them or they have just come out of the hospital or whatever the reason they have phoned in for or come in to see us. We try and ask why they wanted to stop because that is the motivator; then as a lead, once you have got the why; then you discuss the habits around smoking and work out their dependency on nicotine by doing what is called the Fagerstrom test, and it is just part of building a relationship with the person. It is not saying what you smoked 20 a day before 10 a.m.; it is finding out how their day works, assessing how they are not so much mentally but what is going on in their life, have they got family and friends supporting them or are they somebody who is perhaps living on their own and sat there with nothing else to do all day other than smoke,. You can get an idea then of how difficult or how possible the quits will be.*

They believe that therapeutic relationships with people are a foundation for success in quitting smoking; therefore, this would enable them to establish other issues in their lives that may impact their success.

*Fran: If somebody wants to keep returning to the clinic, you see them every week. You get quite a good relationship with people, and you would always find out if they smoked five cigarettes a day this week instead of 20. You find out things that are going on in their personal lives that affect how they will quit and try to give them ideas of what they can do to help them cope with whatever is going on in their lives.*

Advisors emphasised the importance of designing the programme around the user’s personal and social circumstances. They understand that some users may have difficulties accessing the service during typical working hours due to transport, work commitments or childcare. They found that telephone services are particularly useful for users who have work commitments.

In line with the government’s swap-to-stop campaign, advisors present service users with various addiction treatment products, including swapping tobacco products with vaping. They revealed that a mismatch between individual and product could lead to noncompliance and damage clients’ confidence.

*Alice: it is important to match the products with the right patient; it’s got to be if somebody has something set in their mind, they will have that confidence in it; you try to steer them to something different if it’s not suitable for medical reasons.*

Some advisors have expressed reservations about promoting vaping.

*Beth: I was always very weary of e-cigarettes because they weren’t tested. So, I would never want to recommend something that hasn’t been tested because I want to recommend something I would be happy and comfortable using. Now, because we’ve got the public health backing and we’ve got the testing on e-cigarettes, I’m quite comfortable to say to people, this is our guidance from public health; they are 95% less harmful than cigarettes, but we do not know about long-term use, so it is harm reduction.*

The advisor observed that vaping is popular amongst the seldom heard population. Therefore, they must modify their messages from advising against its use to advising them to buy it from reputable sources.

*Beth: Many people in our “hard-to-reach” communities like to vape, especially e-cigarettes, so it is just a matter of getting the message to them that you should go to a reputable seller, do not buy from cabins, and get them from reputable sellers.*

## Discussion

This study aimed to use interpretive phenomenology and socioeconomic determinants theories to analyse the experiences of stop-smoking advisors in promoting smoking cessation initiatives within a disadvantaged neighbourhood in northwest England. This approach provided a unique theoretical understanding of how the neighbourhood affected smoking behaviour. However, approaching the data with ideologically bound frameworks risked taking a biased view of the data. Participants did not always use the term “health’ inequalities” explicitly. Instead, they described the differences between the deprived and the least deprived without directly referencing health inequalities. However, we interpreted their descriptions as relating to health inequalities. Thus, our findings reflected a nuanced interpretation of the meanings that participants attributed to their experiences and practices within their specific contexts.

The central argument of this study was that smoking habits had been higher among communities with socioeconomic deprivation, and traditional behavioural approaches had been less effective in reducing smoking habits in these communities. Therefore, the dual approach that combined behavioural change approaches and societal structural reforms addressed the causes of neighbourhood deprivation.

The argument was supported by data in themes (Table 3) in which participants described typical problems the users in their deprived communities tended to present with, including mental health disorders, unemployment, precarious housing and financial struggles. Arguing that because of the changing nature of their typical users they have had to change their training to incorporate societal theories. These findings were strategically framed around the crucial distinction between ‘upstream’ interventions focusing on society, social institutions and policy‐level determinants, such as income, education, housing, environment and crime, and ‘downstream’ interventions focused on individual factors, increasing access to stop-smoking programmes, reducing attractiveness of tobacco and vaping products and limiting the availability of tobacco and vaping products [62–69]. This distinction was key to understanding the multifaceted nature of smoking cessation strategies [27, 67–70]. This study’s findings were significant in the context of smoking cessation. It underscored the importance of taking a balanced approach that combined downstream individual lifestyle-focused and upstream population-focused stop-smoking interventions in deprived communities [71].

The advisors characterised people who smoke who presented with smoking associated wider social and mental health problems as entrenched, hardened, and hardcore arguing that addressing their unique and complex needs requires more than generic individual lifestyle stop-smoking skills. We interpreted their depiction as a recognition of the uniqueness of these individuals from any other people who smoke. This understanding acknowledges the impact of the social context in which smoking took place as the primary factor influencing smoking, surpassing individual choice. We understood their assertion that the “requires more than generic individual lifestyle stop-smoking skills” to mean that the social context-oriented intervention would be more appropriate than individual-oriented ones. To our knowledge, no published studies have targeted socioeconomic determinants as part of a multifaceted cessation programme for people who smoke in deprived communities.

In this study, advisors noted that people who smoke often encountered challenges associated with poverty, including social isolation, inadequate housing, and financial difficulties. Previous research concurred with these findings, indicating that factors such as employment status, housing conditions, poverty, and residential stability at the community level were independently associated with smoking [72–74]. Research also showed that people who smoke experiencing financial stress were less likely to quit, and if they did quit, they were more likely to relapse [74]. Siahpush’s study further illustrated that people who smoke experiencing stress were more likely to smoke more, leading to a sense of lack of control [72]. Wilkinson and Pickett demonstrated that material deprivation could lead to low self-efficacy in quitting smoking or maintaining a smoke-free behaviour, reducing the likelihood of successful cessation [74, 75]. These issues might not be effectively addressed using traditional smoking cessation methods, and advisors trained in conventional behavioural change techniques might lack the necessary skills to implement interventions targeting the underlying causes of smoking. Considering the strong evidence demonstrating the protective effects of income against smoking, it would be advantageous to incorporate measures that focus on structural approaches that address education and income in deprived communities. [70, 72, 75–77].

In this study, advisors observed that individuals from disadvantaged communities often engaged in multiple health-harming behaviours, such as smoking, alcohol misuse, and diet-related behaviours. Some of these behaviours required more immediate attention than quitting smoking. For example, an advisor recalled a woman who spent the entire session discussing her plan to drink a bottle of vodka that evening. In this case, intervention to reduce the harmful effects of alcohol was more urgent than addressing smoking. However, advisors were more equipped to deal with smoking behaviour than alcohol harm reduction. This highlighted the significance of integrating a broader range of skills in training programmes beyond solely behaviour-oriented methods. This became increasingly relevant as each issue was resolved, often leading to the emergence of new challenges that also had to be addressed.

Advisors provided flexible stop-smoking programs in both traditional and nontraditional healthcare settings to accommodate the specific needs of users. The main objective of these programs was to reduce health inequalities by lowering smoking rates in underserved communities. However, advisors noted that contrary to this goal, the tailored approaches often provided greater benefits to the least deprived individuals than those most deprived. Tudor Hart referred to this phenomenon as the “inverse care law,” which suggested that interventions may not have reached those who needed them the most [21, 27].

In alignment with the UK government’s 2019 recommendation to include e-cigarettes as part of harm reduction strategies for those who smoke and struggle to quit, advisors are offering various addiction treatment options [78]. This includes the replacement of tobacco products with vaping. While some advisors have concerns about promoting vaping, they acknowledge its popularity among underserved populations and adjust their messaging to encourage users to purchase e-cigarettes from reputable sources.

Numerous studies have assessed the effectiveness of e-cigarettes in reducing smoking rates and mitigating associated harms [79, 80]. Research indicates that people who smoke living in deprived communities experience significantly less benefit from the harm reduction effects of e-cigarettes compared to those in least deprive communities [80–82]. One contributing factor is that individuals in deprived communities are more likely to believe that vaping is more harmful than smoking traditional cigarettes, which results in lower usage rates of e-cigarettes in these populations [80, 81].

These disparities further support Tudor Hart’s theory, which suggests that individual-level interventions can increase health inequalities by providing greater benefits to less deprived individuals compared to those in more deprived communities [21].

## Reflexivity

The principal investigator developed the research concept, conducted interviews, and analysed the data. With expertise in the socioeconomic determinants of health inequalities, qualitative research, epidemiology, and biostatistics, their work on social justice and has been widely published in international peer-reviewed journals and books [65, 83, 84].

In various publications and presentations, the researchers articulate how their sociocultural background shaped their perspective on public health as a means to reduce health inequalities. This perspective guided the literature review, data collection, and analysis, emphasising socioeconomic determinants and social justice [65, 83–86].

The study employed an interpretive phenomenological approach, utilising a socioeconomic determinants framework for data analysis and interpretation. Therefore, the findings reflect the researchers’ interpretations of the role of stop-smoking advisors within their specific context.

The researchers outlined the values, beliefs, concepts, and frameworks that influenced their data analysis. While they acknowledge the potential for perceived bias by incorporating the socioeconomic determinants framework, they assert that this reflective approach enhances the study’s transparency and allows for critical assessment of its validity.

With over twenty-year history of conducting qualitative research within this framework, the influence on data interpretation is inherent. Their commitment to transparency and trustworthiness leads to more robust and credible research outcomes.

## Strengths and limitations

The study utilises interpretive phenomenology, which enables a deep understanding of the experiences and perspectives of stop-smoking advisors. This qualitative approach offers valuable insights that are often overlooked by quantitative data, highlighting the complexities of smoking behaviours in deprived communities.

This research addresses a significant gap in the existing literature by emphasising the socioeconomic factors influencing smoking behaviours. It highlights that smoking cessation is not just an individual issue but is deeply connected to the broader social context, underscoring the importance of structural factors alongside individual behaviour changes.

The research is specifically based in a disadvantaged neighbourhood in northwest England, ensuring that the findings are relevant and tailored to the community’s unique needs.

The study identifies four key themes essential for delivering effective stop-smoking services. These themes, which emerged from the experiences and perspectives of stop-smoking advisors, provide a framework for practitioners and policymakers to design interventions that are more responsive to the needs of individuals in deprived areas.

This study has three notable limitations. Firstly, the analysis is conducted through a socioeconomic determinants’ lens, which may make the interpretations susceptible to the researchers’ personal biases and perspectives. As a result, it is possible that different analysts could reach different conclusions from the same dataset based on their own worldviews.

Secondly, the phenomenological approach used in this study suggests that the meanings assigned to participants’ experiences are primarily contextual. Therefore, the findings may only be relevant within their specific contexts, which limits their applicability in different situations.

Lastly, the relatively small sample size raises concerns about the study’s representativeness. However, in addition to the qualitative data collected, the research also included five months of observational data detailing interactions between participants and clients. These observations provided valuable insights that enhanced our understanding of the participants’ experiences.

## Conclusion

In conclusion, the evidence presented indicates that behaviour-oriented interventions have resulted in a more significant decline in smoking rates among the least deprived communities compared to their most deprived counterparts. This phenomenon is commonly called “the inverse care law,” which posits that individuals in greatest need of healthcare services are often the least likely to access them.

To address this issue, policymakers and smoking cessation practitioners are focusing on implementing targeted services in deprived areas. They enhance the workforce’s skills to recognise and communicate the interplay of social, economic, physical, and mental health determinants influencing smoking behaviour.

Furthermore, policymakers and practitioners are adapting their strategies to include various measures, including the use of vaping, as essential components of comprehensive smoking intervention programs tailored to meet the specific needs of vulnerable populations.

The study emphasises the need for both behavioural and structural interventions to address smoking in deprived neighbourhoods, highlighting the influence of socioeconomic factors and the limitations of individual-focused initiatives. To effectively support people who smoke in these areas, interventions should consider broader socioeconomic determinants like social context and material deprivation.

The authors call for government’s efforts to not only target individual behaviour change but also address underlying social conditions. Recommended initiatives include creating high-income job opportunities, improving education, and increasing access to affordable housing. Overall, the study advocates for a comprehensive approach that combines behavioural interventions with strategies to tackle the root causes of smoking in deprived communities.

## Supplementary Information

Supplementary Material 1.

## Data Availability

The datasets generated during and/or analysed during the current study are available from the corresponding author on reasonable request.
