# Supplementary material for: Exploring the social context of smoking behaviours: insights from stop-smoking advisors in deprived communities in Northwest of England UK
Source: BMC Public Health. 2025 May 23;25:1914. doi: 10.1186/s12889-025-23110-7 (PMC12100904; doi:10.1186/s12889-025-23110-7)
Supplement: Supplementary file 1 — Supplementary Material 1. [file 12889_2025_23110_MOESM1_ESM.docx]

**Appendix 7**

**Interview Schedule/Topic Guide**

Welcome, introductions, and housekeeping, such as a fire alarm sounding, directions to the nearest toilets, refreshment facilities, and quiet areas if the participant needs a break from the interview.

The researcher and participant are to complete the consent form.

Remind the participant of their right to withdraw from the study, either temporarily or permanently, without the need to provide a reason and without consequence for doing so. Emphasise that the participant may withdraw from the study at any time before or during the interview and for up to one week following member checking; the withdrawal of the participant's data will not be possible after this time.

Check permission to record the interview and make field notes.

Give a brief overview of the nature and purpose of the research study.

Provide a verbal outline of the interview schedule and advise the participant that they can take a break at any time during the interview. Also, advise the participant that if they are affected by the nature, content, and progression of the interview, they may request that the interview be stopped and that support, guidance, and advice be sought.

Advise the participant of the measures in place to ensure confidentiality and anonymity throughout the study.

Advise the participant of the member checking process that will be facilitated during the data analysis stage of the study.

Express thanks to the participant for agreeing to take part in the study.

Invite participants to provide a brief overview of their healthcare background, experience as a stop-smoking advisor, and academic credentials.

The researcher will use the research questions to guide but not direct the interview to allow for the exploration of responses and unanticipated themes: -

- Please tell me about your experiences of providing stop-smoking advice in deprived communities.
- Please tell me how to ensure that you feel empowered to undertake this role
- Can you explain how this team is led and team dynamics
- What are your interpretations of productive work
- What are your perceptions of the knowledge, skills, competencies, and confidence needed to facilitate smoking cessation?

Ask the participant if there is anything else they would like to add or say.

- Ask the participants if they have any questions.
- Thank the participant for taking part in the interview.
- Give the participant the researcher’s contact details.
